# Risk Factors for Postoperative Complications Following Resection of Colorectal Liver Metastases and the Impact on Long-Term Survival: A Population-Based National Cohort Study

**DOI:** 10.1007/s00268-023-07043-z

**Published:** 2023-05-20

**Authors:** Peter Scherman, Ingvar Syk, Erik Holmberg, Peter Naredi, Magnus Rizell

**Affiliations:** 1 Department of Surgery, Institute of Clinical Sciences, Sahlgrenska Academy, University of Gothenburg, Gothenburg, Sweden; 2 Department of Surgery, Helsingborg Hospital, Charlotte Yhlens gata 10, 254 37 Helsingborg, Sweden; 3 Department of Surgery, Clinical Sciences Malmö, Lund University, Lund, Sweden; 4 Department of Surgery, Skåne University Hospital, Malmö, Sweden; 5 Department of Oncology, Institute of Clinical Sciences, Sahlgrenska Academy, University of Gothenburg, Gothenburg, Sweden; 6 Department of Surgery, Sahlgrenska University Hospital, Gothenburg, Sweden; 7 Department of Transplantation, Sahlgrenska University Hospital, Gothenburg, Sweden

## Abstract

**Background:**

Postoperative complications (POCs) following resection of colorectal liver metastases (CRLM) are common. The objective of this study was to evaluate risk factors for developing complications and their impact on survival considering prognostic factors of the primary tumor, metastatic pattern and treatment in a well-defined national cohort.

**Methods:**

Patients treated with resection for CRLM that was also radically resected for their primary colorectal cancer (diagnosed in 2009–2013) were identified in Swedish national registers. Liver resections were categorized according to extent of surgery (Category I–IV). Risk factors for developing POCs as well as prognostic impact of POCs were evaluated in multivariable analyses. A subgroup analysis of minor resections was performed to evaluate POCs after laparoscopic surgery.

**Results:**

POCs were registered for 24% (276/1144) of all patients after CRLM resection. Major resection was a risk factor for POCs in multivariable analysis (IRR 1.76; *P* = 0.001). Comparing laparoscopic and open resections in the subgroup analysis of small resections, 6% (4/68) in the laparoscopic group developed POCs compared to 18% (51/289) after open resection (IRR 0.32; *P* = 0.024). POCs were associated with a 27% increased excess mortality rate (EMRR 1.27; *P* = 0.044). However, primary tumor characteristics, tumor burden in the liver, extrahepatic spread, extent of liver resection and radicality had higher impact on survival.

**Conclusion:**

Minimal invasive resections were associated with a decreased risk of POCs following resection of CRLM which should be considered in surgical strategy. Postoperative complications were associated with a moderate risk for inferior survival.

**Supplementary Information:**

The online version contains supplementary material available at 10.1007/s00268-023-07043-z.

## Introduction

Approximately 25% of colorectal cancer patients develop liver metastases (CRLM) within three years of diagnosis [1] and surgical resection or ablation of CRLM is increasingly common [2, 3]. Although indications have widened, population-based outcome studies have demonstrated that a 5-year relative survival (RS) of approximately 50% is expected after liver resection [3, 4]. To further improve prognosis, surgical strategies aimed to extend the treatable cohort of CRLM are proposed, but the risks associated with surgical approach and extent of surgery must be considered. The importance of postoperative complications (POCs) for survival is relevant and extensive surgery is challenged by alternatives to open resection. Among those, ablation therapy is increasingly used to minimize patient morbidity, but long-term results have been questioned [5, 6].

The randomized Oslo-COMET trial showed a decreased complication rate and faster recovery following laparoscopic resections [7]. Consensus documents advocate the use of a risk score strategy when introducing the laparoscopic technique, thereby avoiding difficult segments and extensive resections [8, 9]. In Sweden, all six liver centers practice enhanced recovery protocols after liver surgery and although it is still a minority of CRLM cases [10] an increasing number of cases are performed by laparoscopic surgery.

To date, knowledge on risk factors for complications following CRLM resection are scarce, but POCs have been associated with subsequent impaired long-term results [11, 12]. An awareness of risk factors and their implications for prognosis might impact treatment strategies. A lower risk of complications might increase the potential for treating high risk patients such as the elderly and patients with co-morbidity, where there is an increased need [4, 13]. The objectives of this study were therefore: (1) to map POCs in a cohort of patients with CRLM where risk factors depending on the primary tumor and surgery were known; (2) to identify risk factors for complications; and (3) to evaluate their impact on survival.

## Methods

Data were collected in February 2018 from two national registries with prospectively registered data, i.e. the Swedish Colorectal Cancer Registry (SCRCR) and the National Quality Registry for Liver Cancer (SweLiv). Both registries have reported good conformity with source data throughout the study period and high coverage when compared to the Swedish Cancer Registry [10, 14–16]. Patients radically treated for their primary colorectal cancer between 2009 and 2013 and subsequently treated for CRLM between 2009 and 2016 were identified [4]. If more than one liver surgery event was registered, only the first was accounted for. The study cohort comprises patients treated with resection (with or without simultaneous ablation therapy) and the complete registration of POCs within 30 days. Patients with postoperative complications after the primary colorectal surgery treated for liver metastases within 30 days were excluded. Follow-up data were updated in March 2019 and survival was crosschecked with the Swedish Population Register. This study was approved by the Regional Ethical Review Board in Gothenburg (No. 189–15). Patient registries inform patients of the use of data for study purposes. No further informed consent was required for this study.

### Postoperative complications

All POCs classified as Clavien–Dindo IIIa or worse were included but POCs were not registered according to the Clavien–Dindo classification before 2013. The following registered complications were therefore included for the entire study period (2009–2016): bile leakage, wound dehiscence, postoperative bleeding and other surgical complications demanding intervention or reoperation, single organ failure including liver failure, renal failure demanding dialysis*,* heart infarction and cerebral infarction, ascites or pleural fluid demanding intervention, deep vein thrombosis or pulmonary embolism, portal vein thrombosis, postoperative intensive care more than 24 h, intra-abdominal infections demanding intervention, sepsis and other infections demanding intervention or reoperation. Special importance of infectious POCs has been proposed [17, 18] and established pneumonia demanding antibiotic treatment was therefore included.

### Extent of resection

Based on SweLiv data of resected segments validated with surgical procedure codes, liver resections were categorized into four groups depending on the extent of surgery; i.e.*,* anatomical resection of one segment or 1–2 wedge resections (Category I); anatomical resection of two segments or 3–4 wedge resections (Category II); anatomical resections including more than 2 segments but not including hemihepatectomies or extended resections, or in case of wedge resections more than 4 wedge resections (Category III); and all hemihepatectomies including extended hemihepatectomies and two-step resections (Category IV). Two-step resections were defined as two surgical events within 6 weeks.

### Statistical analysis

All statistical analyses were performed using Stata version 16.1 (StataCorp, College Station, Texas, USA). A *P*-value of < 0.05 was considered statistically significant. Risk factors for POCs were analyzed using Poisson regression and presented as incidence rate ratios (IRR) with 95% confidence intervals. Survival was calculated from the date of liver surgery to date of death or date of last follow-up (21st March 2019). Overall survival (OS) was computed using the Kaplan–Meier method and RS was calculated using the Ederer II method [19]. Age-standardization of RS was performed using the standard weight distributions for cancers (ICSS 1 standard) [20]. Mortality rates by sex, 1-year age group and 1-year calendar period for the general population in Sweden were used to estimate expected survival rates for the study populations. The relative risk between different groups for excess mortality rate was analyzed using Poisson regression analysis and is presented as the excess mortality rate ratio (EMRR) with 95% confidence interval [21]. Variables that were significant at *P* < 0.10 in univariable analyses together with previously established statistically significant prognostic factors were tested in multivariable analyses through manual elimination to assess their confounding effect or independent effect on POCs and excess mortality.

The following prognostic factors, previously found to influence patient survival [5], were included in multivariable analyses of risk factors for POCs as well as excess mortality: age, sex, ASA-score, Lymph node ratio (LNR), tumor grade, vascular invasion, acute/elective primary surgery, and severe postoperative complications after primary surgery, lung or other metastases before or at liver intervention and response to chemotherapy. The number of tumors and size of metastases were replaced with the extent of liver resection. Anatomical or non-anatomical resection, microscopic radicality and bleeding were included in multivariable analyses in addition to the previously described prognostic factors. Laparoscopic or open resection was included in the survival analyses but not when analyzing the risk of POCs due to co-variation with the extent of resection. To evaluate POCs after laparoscopic surgery compared to open resections, a subgroup analysis of small resections suitable for laparoscopic techniques was performed (≤ 2 metastases with largest metastasis ≤ 50 mm categorized as only anterolateral (segment 2, 3, 4b, 5 and/or 6) or only posterosuperior (segment 1, 4a, 7 and/or 8)).

## Results

Out of the 20,853 colorectal cancer patients treated with radical resection between 2009 and 2013, a total of 1200 (5.8%) patients were also registered in SweLiv for resection of liver metastases. Of these, complication data after liver resection were registered for 1166 (97.3%) patients. Twenty-two patients treated for CRLM within 30 days of the primary surgery were excluded due to registered postoperative complications after colorectal surgery in SCRCR. Hence, 1144 patients constitute the study cohort, of which 733 (64.1%) patients were treated with chemotherapy before liver surgery. Open resection was performed in 957 (83.7%) patients, 91 (8.0%) patients were treated with laparoscopic resection and 96 (8.4%) patients with resection (open or laparoscopic) combined with ablation therapy. Major resection (Category IV) including 18 (1.6%) two-stage procedures was performed in 444 (38.8%) patients. Details on treatment are presented in Table 1.

**Table 1:** Descriptive data of resections of colorectal cancer liver metastases (2009–2016) stratified on extent of resection (Category I–IV)

	All resections N = 1144†	Category I n = 349 (30.1%)	Category II n = 276 (24.1%)	Category III n = 73 (6.4%)	Category IV n = 444 (38.9%)
Age (years) *	66 (25–87)	67 (33–87)	67 (25–86)	65 (35–83)	66 (35–85)
*Sex*					
Males	698 (61.0)	219 (62.8)	169 (61.2)	41 (56.2)	268 (60.4)
Females	446 (39.0)	130 (37.2)	107 (38.8)	32 (43.8)	176 (39.6)
*ASA*					
1–2	897 (78.4)	274 (78.5)	209 (75.7)	59 (80.8)	353 (79.5)
3–4	239 (20.9)	74 (21.2)	65 (23.6)	14 (19.2)	86 (19.4)
Missing	8 (0.7)	2 (0.7)	2 (0.7)	0	5 (1.1)
Chemotherapy before surgery	733 (64.1)	147 (42.1)	156 (56.5)	62 (84.9)	366 (82.4)
Missing	3 (0.3)	0	2 (0.7)	0	1 (0.2)
Portal vein embolization	33 (2.9)	1 (0.3)	0	0	1 (0.2)
Missing	4 (0.4)	1 (0.3)	2 (0.7)	0	
Two-stage procedure	18 (1.6)	0	0	0	18 (4.0)
Liver first	187 (16.4)	27 (7.7)	36 (13.0)	26 (35.6)	98 (22.1)
*Type of surgery*					
Anatomical	433 (37.9)	73 (20.9)	94 (34.1)	12 (16.4)	253 (57.0)
Non-anatomical	368 (32.2)	267 (76.5)	69 (25.0)	20 (27.4)	12 (2.8)
Both	312 (27.3)	–	107 (38.8)	40 (54.8)	164 (36.9)
Missing	31 (2.7)	9 (2.6)	6 (2.2)	1 (1.4)	15 (3.4)
*Type of surgery*					
Open resection	957 (83.7)	272 (77.9)	215 (77.9)	60 (82.2)	408 (91.9)
Laparoscopic resection	91 (8.0)	52 (14.9)	37 (13.4)	1 (1.4)	1 (0.2)
Resection + ablation	96 (8.4)	25 (7.2)	24 (8.7)	12 (16.4)	35 (7.9)
*Perioperative bleeding (ml)*					
0–199	171 (15.0)	89 (25.5)	50 (18.1)	8 (11.0)	24 (5.4)
200–449	319 (27.9)	128 (36.7)	79 (28.6)	14 (19.2)	98 (22.1)
450–999	299 (26.1)		78 (28.3)	14 (19.2)	128 (28.8)
1000-	332 (29.0)	78 (22.4)	62 (22.5)	35 (48.0)	190 (42.8)
Missing	23 (2.0)	44 (12.6)10 (2.9)	7 (2.5)	2 (2.7)	4 (0.90)
*Radicality*					
R0 (surgeons’ opinion)	1023 (89.6)	319 (91.4)	251 (90.9)	59 (80.8)	394 (88.7)
R1/uncertain	117 (10.2)		24 (8.7)	14 (19.2)	30 (11.3)
Missing	2 (0.2)	29 (8.3)	1 (0.4)	0	0
R0 (histopathology)	913 (80.0)	1 (0.9)	232 (84.1)	52 (71.2)	338 (76.1)
R1/uncertain	172 (15.0)	291 (83.4)	33 (12.0)	18 (24.7)	83 (18.7)
Missing	57 (5.0)	38 (10.9)20 (5.7)	11 (4.0)	3 (4.1)	23 (5.2)

Values in parentheses are percentages unless indicated otherwise. Category I: Anatomical resection of 1 segment or 1–2 wedge resections. Category II: anatomical resection of 2 segments or 3–4 wedge resections. Category III: anatomical resections of > 2 segments or 4 wedge resections. Category IV: all hemihepatectomies including extended resections

*Values in parentheses are median (range). **Values in parentheses are 95% confidence intervals. †Two patients were not classified according to extent of resection.

### Postoperative complications

**Fig. 1:**
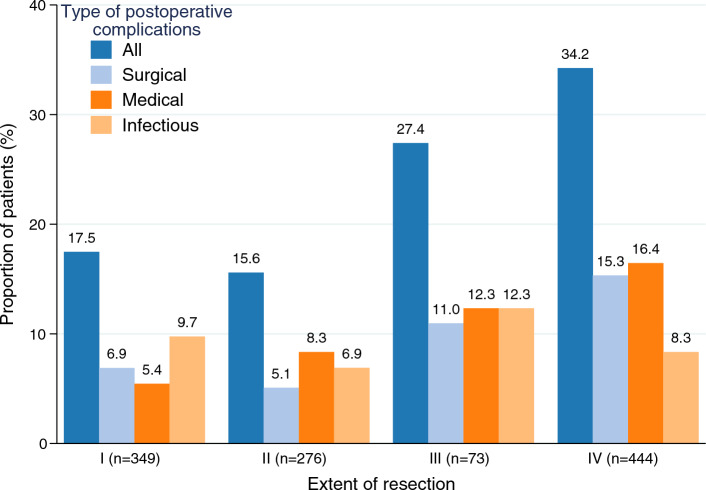
Postoperative complications following resection of colorectal cancer liver metastases according to extent of resection. Postoperative complications including Clavien–Dindo IIIa or worse and including established pneumonia treated with antibiotics, thromboembolic events and intensive care > 24 h

Postoperative complications corresponding to CD IIIa or more were registered for 276 (24.1%) whereof five patients died within 30 days of surgery (0.44%). Surgical complications, including bile leakage, wound dehiscence, and postoperative bleeding, were registered for 114 (10.0%) patients. Medical complications including single organ failure, ascites or pleural fluid demanding intervention and venous thromboembolism were registered in 124 (10.8%) patients and infectious complications in 99 (8.7%) patients. Surgical and medical complications were increasingly common when more extensive resections were performed (*P* < 0.01), whereas infectious complications were not (Fig. 1; Table 2). In the multivariable analysis, only major resection (Category IV) was a significant risk factor (IRR 1.76, 95% CI 1.26–2.46; Table 3)*.* Hospital stay in days was registered for 92.0% (1052/1144) of the patients and patients with POCs had significantly longer median hospital stays (11 days) compared to patients with no POCs (8 days, *P* < 0.001).

**Table 2:** Type of postoperative complications within 30 days following resection of colorectal cancer liver metastases stratified on extent of resection

	All resections N = 1144†	Category I n = 349	Category II n = 276	Category III n = 73	Category IV n = 444	*P*^1^
Surgical complications*	114 (10.0)	24 (6.9)	14 (5.1)	8 (11.0)	68 (15.3)	< 0.001
Bile leakage	70 (6.1)	11 (3.2)	6 (2.2)	5 (6.8)	48 (10.8)	< 0.001
Wound dehiscence	11 (1.0)	2 (0.6)	3 (1.1)	0 (0.0)	6 (1.4)	0.72
Postoperative bleeding	13 (1.1)	3 (0.9)	1 (0.4)	1 (1.4)	8 (1.8)	0.29
Other surgical complications	37 (3.2)	10 (2.9)	7 (2.5)	2 (2.7)	18 (4.1)	0.72
Medical complications**	124 (10.9)	19 (5.4)	23 (8.3)	9 (12.3)	73 (16.4)	< 0.001
Liver failure	17 (1.5)	1 (0.3)	2 (0.7)	0 (0.0)	14 (3.2)	0.005
Renal failure	3 (0.3)	0 (0.0)	1 (0.4)	1 (1.4)	1 (0.2)	0.18
Ascites or pleural fluid	64 (5.6)	12 (3.4)	9 (3.3)	4 (5.5)	39 (8.8)	0.003
Venous thromboembolism	38 (3.3)	5 (1.4)	9 (3.3)	3 (4.1)	21 (4.7)	0.055
Other medical complications	13 (1.1)	2 (0.6)	4 (1.4)	2 (2.7)	5 (1.1)	0.30
Infectious complications**	99 (8.7)	34 (9.7)	19 (6.9)	9 (12.3)	37 (8.3)	0.38
Intra-abdominal infections	40 (3.5)	14 (4.0)	6 (2.2)	5 (6.8)	15 (3.4)	0.23
Sepsis	29 (2.5)	10 (2.9)	4 (1.4)	2 (2.7)	13 (2.9)	0.59
Other infections	49 (4.3)	17 (4.9)	11 (4.0)	4 (5.5)	17 (3.8)	0.79
30 days mortality	5 (0.44)	0 (0.0)	2 (0.72)	1 (1.4)	2 (0.45)	0.19
All complications	276 (24.1)	61 (17.5)	43 (15.6)	20 (27.4)	152 (34.2)	< 0.001

Values in parentheses are percentages unless indicated otherwise. †Two patients were not classified according to extent of resection. *Surgical complications (yes/no) were registered in Sweliv for all patients. **Registration of medical/infectious complications (yes/no) were missing for 7 patients. ^1^Fisher´s exact test. Category I: Anatomical resection of 1 segment or 1–2 wedge resections. Category II: anatomical resection of 2 segments or 3–4 wedge resections. Category III: anatomical resections of > 2 segments or 4 wedge resections. Category IV: all hemihepatectomies including extended resections

**Table 3:** Risk factors for postoperative complications within 30 days following resection of colorectal cancer liver metastases

	N = 1144	Number of POCs	Univariable		Multivariable	
			Poisson regression		Poisson regression†	
			IRR*	P	IRR*	P
*Sex*						
Male	698 (61.0)	183 (26.2)	Ref			n.s
Female	446 (39.0)	93 (20.9)	0.80 (0.62–1.02)	0.072		
*Age*					Not included	
<60	300 (26.2)	68 (22.7)	Ref			
60–74	613 (53.6)	151 (24.6)	1.09 (0.82–1.45	0.569		
≥75	231 (20.2)	57 (24.7)	1.09 (0.77–1.55)	0.636		
*ASA*					Not included	
1–2	897 (78.4)	213 (23.8)	Ref			
3–4	239 (20.1)	61 (25.5)	1.07 (0.81–1.43)	0.619		
Missing	8					
Preop chemo **						n.s
No	408 (35.7)	84 (20.6)	Ref			
Yes	733 (64.1)	192 (26.2)	1.27 (0.98–1.64)	0.066		
Missing	3					
*Metastatic pattern (at time of liver treat.)*					Not included	
Only liver met	906 (79.2)	213 (23.6)	Ref			
Liver and lung met	171 (14.9)	46 (26.9)	1.14 (0.83–1.57)	0.407		
Liver and other met	67 (5.9)	17 (25.4)	1.08 (0.66–1.77)	0.762		
Missing	50 (4.4)					
*Type of resection*						
Anatomical	433 (37.8)	103 (23.8)	Ref		Not included	
Non-anatomical	368 (32.3)	77 (20.1)	0.88 (0.65–1.18)	0.395		
Both	312 (27.3)	89 (28.5)	1.20 (0.99–1.59)	0.209		
Missing	31					
*Extent of resection*						
Category I	349 (30.5)	61 (17.5)	Ref		Ref	
Category II	276 (24.1)	43 (15.6)	0.89 (0.60–1.32)	0.564	0.86 (0.57–1.31)	0.490
Category III	73 (6.4)	20 (27.4)	1.57 (0.95–2.60)	0.081	1.51 (0.89–2.55)	0.128
Category IV	444 (38.8)	152 (34.2)	1.96 (1.46–2.64)	<0.001	1.76 (1.26–2.46)	0.001
Missing	2					
*Histopathology*						
Radical (R0)	915 (80.0)	204 (22.3)	Ref		Ref	
Non-radical (R1)	172 (15.0)	56 (32.6)	1.46 (1.09–1.96)	0.012	1.31 (0.97–1.76)	0.080
Missing	57					
*Bleeding (ml)*						
0–199	171 (14.9)	26 (15.2)	Ref		Ref	
200–449	319 (27.9)	63 (19.8)	1.30 (0.82–2.05)	0.262	1.14 (0.71–1.83)	0.589
450–999	299 (26.1)	72 (24.1)	1.58 (1.01–2.48)	0.044	1.34 (0.84–2.13)	0.224
>1000	332 (29.0)	109 (32.8)	2.16 (1.41–3.31)	<0.001	1.56 (0.99–2.47)	0.058
Missing	23					

Values in parentheses are percentages unless indicated otherwise. *Values in parentheses are 95% confidence intervals. **Adjuvant to primary tumor surgery or neoadjuvant treatment before liver resection. †Number of tumors, tumor size and type of treatment (laparoscopic/open resection) were not included in multivariable analysis due to covariation with extent of surgery. Surgical complications (yes/no) were registered in Sweliv for all patients. Registration of medical/infectious complications (yes/no) were missing for 7 patients. The following primary tumor variables were not statistically significant (*P* < 0.1) in univariable analysis and not included in multivariable analysis: Lymph node ratio, differentiation grade, vascular invasion, acute or elective surgery and severe complication after primary surgery. POCs, postoperative complications; IRR, incidence rate ratio

The subgroup analysis designed to include cases suitable for laparoscopic resection (i.e., ≤ 2 metastases, with largest metastasis ≤ 50 mm) included 68 patients treated with laparoscopy and 289 with open resection. There was no significant difference in median tumor size between the laparoscopic (20 mm) and open resection (20 mm) groups (*P* = 0.48). In the laparoscopic group, 5.9% (4/68) of the patients were registered with POCs compared to 17.7% (51/289) in the open resection group. This corresponded to a 68% decreased incidence rate ratio (IRR 0.32; *P* = 0.024) in multivariable analysis adjusted for sex, preoperative oncological treatment, and anatomical or non-anatomical resection. Additional adjustment for anterolateral or posterosuperior resections did not affect the model and was not included in multivariable analysis of the subgroup (Table S1; supporting information). No postoperative bile leakage was registered after laparoscopic resection, while bile leakage was registered for 3.1% (9/289) of the patients after open resection (*P* = 0.22). Postoperative hospital stay was significantly shorter for patients treated laparoscopically (5 days) compared to open resection (8 days; *P* < 0.001)*.*

### Survival

The 5-year OS rate was 51.3% (95% CI 48.3–54.3) following resection of CRLM and the 5-year age-standardized RS rate was 56.1% (95% CI 52.6–59.7; Table 1*)*. In patients with postoperative complications the 5-year RS was 48.8% (95% CI 42.1–58.1) compared to 58.1% (95% CI 54.1–62.3) in cases without complications, corresponding to an increased relative risk of 27% in multivariable analyses (EMRR 1.27, 95% CI 1.01–1.61). All risk factors including primary tumor characteristics and metastatic patterns are presented in Table 4.

**Table 4:** Relative survival and excess mortality rate ratio (EMRR) following resection of colorectal cancer liver metastases

	N = 1144	5-year age-standardized relative survival*	Univariable		Multivariable	
			Poisson regression		Poisson regression	
			EMRR*	*P*	EMRR*	*P*
POCs after liver surgery**						
No	868 (75.9)	58.1 (54.1–62.3)	Ref	0.010	Ref. 1.27	0.044
Yes	276 (24.1)	48.8 (42.1–56.5)	1.33 (1.07–1.65)		(1.01–1.61)	
*Sex*					Not included	
Male	698 (61.0)	52.9 (44.0–63.8)	Ref			
Female	446 (39.0)	61.9 (55.8–68.8)	1.08 (0.89–1.32)	0.426		
*Age*					Not included	
< 60	300 (26.2)	58.1 (52.1–63.6)^#^	Ref			
60–74	613 (53.6)	55.2 (50.7–59.5)^#^	1.08 (0.86–1.34)	0.523		
≥ 75	231 (20.2)	56.4 (47.7–64.8)^#^	1.11 (0.81–1.50)	0.524		
*ASA*					Not included	
1–2	897 (78.4)	57.1 (53.2–61.4)	Ref	0.223		
3–4	239 (20.1)	51.7 (43.7–61.3)	1.16 (0.91–1.48)			
Missing	8					
*Extent of resection*						
Category I	349 (30.5)	67.5 (61.6–73.9)	Ref		Ref	
Category II	276 (24.1)	55.6 (48.8–63.4)	1.47 (1.09–1.99)	0.011	1.50 (1.08–2.07)	0.015
Category III	73 (6.4)	47.0 (35.7–61.8)	2.04 (1.37–3.03)	<0.001	1.66 (1.05–2.61)	0.028
Category IV	444 (38.8)	49.1 (43.6–55.3)	1.88 (1.45–2.44)	<0.001	1.79 (1.33–2.40)	<0.001
Missing	2 (0.2)					
*Type of resection*						
Open resection	957 (83.7)	56.7 (52.9–60.7)	Ref		Ref	
Laparoscopic resection	91 (8.0)	70.1 (58.7–83.7)	0.62 (0.38–1.00)	0.051	0.82 (0.50–1.34)	0.429
Resection + ablation	96 (8.4)	35.5 (26.1–48.2)	1.74 (1.30–2.32)	<0.001	1.64 (1.19–2.27)	0.003
*Histopathology*						
Radical (R0)	915 (80.0)	59.3 (55.4–63.4)	Ref		Ref	
Non-radical (R1)	172 (15.0)	36.2 (28.3–46.3)	1.82 (1.44–2.31)	<0.001	1.58 (1.22–2.05)	<0.001
Missing	57 (5.0)					
*Metastatic pattern (at time of liver treatment)*						
Only liver metastases	906 (79.2)	61.6 (57.8–65.7)	Ref		Ref	
Liver and lung metastases	171 (15.0)	40.4 (32.0–51.1)	1.75 (1.37–2.24)	< 0.001	1.72 (1.31–2.24)	< 0.001
Liver and other metastases	67 (5.9)	17.2 (9.4–31.6)	3.23 (2.37–4.40)	< 0.001	3.32 (2.37–4.66)	< 0.001
Primary tumor variables						
*LNR (lymph node ratio)*						
0	424 (37.1)	68.8 (63.2–74.9)	Ref		Ref	
> 0 to < 0.1	228 (19.9)	59.5 (52.2–67.8)	1.34 (0.99–1.83)	0.060	1.10 (0.78–1.54)	0.587
0.1 to < 0.25	237 (20.7)	50.0 (43.0–58.2)	1.92 (1.45–2.54)	< 0.001	1.61 (1.18–2.20)	0.003
≥ 0.25	289 (21.8)	36.5 (30.2–44.2)	2.62 (2.02–3.41)	< 0.001	2.04 (1.52–2.73)	< 0.001
Missing	6 (0.5)					
*Tumor grade*						
High/mean	935 (82.2)	57.8 (54.0–61.9)	Ref		Ref	
Low	152 (13.4)	41.2 (33.2–51.3)	1.73 (1.34–2.23)	< 0.001	1.59 (1.21–2.09)	0.001
Missing	51 (4.5)					
*Vascular invasion*						
No	629 (55.0)	65.3 (60.8–70.2)	Ref		Ref	
Yes	455 (39.8)	43.2 (38.1–49.1)	1.99 (1.62–2.44)	< 0.001	1.62 (1.29–2.02)	< 0.001
Missing	60 (5.2)					
POCs after primary surgery						
No	888 (77.6)	58.3 (54.4–62.4)	Ref		Ref	
Yes	68 (5.9)	35.9 (24.2–53.4)	1.75 (1.23–2.50)	0.002	1.74 (1.19–2.55)	0.004
N/a (liver first)***	187 (16.4)	48.9 (39.4-60.7)	1.26 (0.99–1.62)	0.063	1.16 (0.88–1.51)	0.293
Missing	1 (0.1)					

Values in parentheses are percentages unless indicated otherwise. POCs, postoperative complications. EMRR, excess mortality rate ratio. ^#^5-year relative survival without age-standardizing

*Values in parentheses are 95% confidence intervals. **Surgical complications (yes/no) were registered in Sweliv for all patients. Registration of medical/infectious complications (yes/no) were missing for 7 patients. ***Liver surgery performed before primary tumor surgery.

## Discussion

In this population-based study, POCs and their impact on survival following resection of CRLM were studied when considering prognostic factors of the primary tumor, metastatic pattern, treatment strategy and outcome variables such as bleeding and radicality. Our results show POCs to be an independent risk factor for impaired long-term survival and support minimal invasive surgery (laparoscopy) to reduce complications and increase survival.

Postoperative complications following liver surgery continue to be a concern. Enhanced Recovery After Surgery (ERAS) seems to be efficient in decreasing hospital stays, but not in preventing complications [22]. In our study cohort, 24% (276/1144) of all patients treated for CRLM developed significant complications. Hospital stay was significantly prolonged by POCs, emphasizing the negative impact not only for patients but also on cost-effectiveness. R1-resection (histopathology) and extensive bleeding (>499 ml) were significant risk factors for developing POCs in univariable analyses, both suggesting difficult or complicated procedures. A majority of patients (64%) had received chemotherapy before liver surgery and there was no significant difference in POC compared to those who were chemotherapy naïve. In multivariable analysis, only the extent of resection was a significant risk factor, with an 87% increased risk for major (Category IV) resections compared to small (Category I) resections. Age did not affect the risk of POCs or long-term survival, supporting earlier conclusions that a selected group of elderly patients benefit from liver surgery, taking patient risk factors, primary tumor and metastatic pattern, into account [4, 13].

By stratifying the liver resections into four groups, where only the fourth would be considered major, we aimed to give a clinical description of our cohort where single and small tumors are common. The definition of major liver resections as three or more liver segments [23] is widely used, and includes resections with high complication rates [24]. It is therefore not surprising that Category IV resections had the highest risk of POCs, and that bile leakage as well as liver failure were mainly seen in this group. However, an increase in both medical and surgical complications was also observed in Category III resections.

R1-resections were seen in 16% (40/276) of Category II resections and 29% (21/73) of Category III-resections, possibly indicating increased complexity in precision as the number of tumors rises. Since complications increase and radicality decreases in Category III, one can infer that multiple minor resections should not be regarded as minor surgery, and that further efforts to allow better precision and thus radicality are needed. Poor survival after R1-resections could indeed be related to unfavorable tumor biology or complications, but the close to 60% increased risk of excess mortality shown here underlines the importance of aiming for radical resections when feasible.

Intraoperative bleeding is still a relevant clinical problem, although low-pressure anesthesia and surgical techniques including Pringle have decreased bleeding volumes [25, 26]. Blood transfusion as a cause of postoperative inflammation and its potential to decrease survival has been debated [27–29]. In this study, the association of bleeding with POCs was only significant in univariable analysis, possibly related to size and complexity of the surgery, and there was no correlation between bleeding and survival.

Others have reported that laparoscopy has the potential to reduce POCs [7, 30, 31]. Our concern was that complication rates might be higher in surgical centers in Sweden, which are less experienced in laparoscopic surgery. Hence, we performed a subgroup analysis of tumors suitable for laparoscopic resections aiming to reduce differences in tumor and technical complexity between the laparoscopic and open groups. The analysis showed a significant reduction in POCs in the laparoscopy group. Furthermore, bile leakage was rare in this laparoscopically resected cohort, which we found especially encouraging. The decreased risk for bile leakage after laparoscopic surgery might not only be a matter of location and size, and one could hypothesize that the technique with magnification, usually slower transection of the parenchyma and use of instruments like LigaSure™, might decrease the risk for bile leakage.

In our study, EMRR is increased after POCs. However, in multivariable analysis, ablation in combination with resection, R1-resection and category II–IV resections were stronger risk factors associated with over 50% higher EMRR. The negative impact of complications on both long-term survival and tumor-free survival is in line with other studies [11, 12, 17].

The strength of this study is the size of the study (n = 1144), and the fact that the impact of complications is studied in the context of other clinical factors known to have a prognostic impact. The most important limitation of this study is that it is retrospective, although data have been prospectively reported in national registries with high coverage. The validity of complication registration between different centers during a long period of time (2009–2016) will be inferior to prospective registrations by trained personnel. However, we have focused this paper on serious complications where the validity is better [32].

In summary, almost one-quarter of all patients undergoing CRLM resection developed severe POCs within 30 days. Postoperative complications were significantly more common after major resections and were associated with both inferior long-term survival and prolonged postoperative hospital stays. Although the noted lower risk of complications in laparoscopic surgery was not translated into a benefit for long-term survival, our results support further development and the increased use of minimally invasive liver surgery for CRLM.

## Supplementary Information

Below is the link to the electronic supplementary material.

Supplementary file1 (DOCX 15 kb)
