# Supplementary material for: Risk Factors for Postoperative Complications Following Resection of Colorectal Liver Metastases and the Impact on Long-Term Survival: A Population-Based National Cohort Study
Source: World J Surg. 2023 May 20;47(9):2230–40. doi: 10.1007/s00268-023-07043-z (PMC10387456; doi:10.1007/s00268-023-07043-z)
Supplement: Supplementary file 1 — Supplementary file1 (DOCX 15 kb) [file 268_2023_7043_MOESM1_ESM.docx]

**Supplementary table 1.** Subgroup analysis of risk factors for postoperative complications within 30 days following resection of colorectal cancer liver metastases

|  | **N=357** | **Univariable  Poisson regression** | | **Multivariable  Poisson regression** | |
| --- | --- | --- | --- | --- | --- |
|  |  | **IRR*** | ***P*** | **IRR*** | ***P*** |
| **Type of surgery**  Open resection  Laparoscopy | 289 (81.0)  68 (19.0) | Ref.  0.33 (0.12–0.89) | 0.029 | Ref.  0.32 (0.12–0.86) | 0.024 |
| **Sex**  Male  Female | 220 (61.6)  137 (38.4) | Ref.  0.60 (0.35–1.05) | 0.073 | Ref.  0.64 (0.37–1.12) | 0.118 |
| **Type of resection**  Anatomical  Non-anatomical  Both | 127 (35.6)  207 (60.0)  23 (6.4) | Ref.  1.71 (0.97–3.02)  0.79 (0.19–3.25) | 0.065  0.743 | Ref.  1.60 (0.91–2.81)  0.82 (0.19–3.49) | 0.103  0.789 |
| **Preop. chemotherapy**  No  Yes | 215 (60.2)  140 (39.8) | Ref.  0.52 (0.30–0.93) | 0.026 | Ref.  0.54 (0.30–0.96) | 0.037 |
| **Location**  Anterolateral  Posterosuperior | 237 (66.4)  120 (33.6) | Ref.  1.04 (0.63–1.74) | 0.874 | Not included |  |

Values in parentheses are percentages unless indicated otherwise. *values in parentheses are 95% confidence intervals. RS, relative survival. IRR, incidence rate ratio. Subgroup analysis with ≤2 liver metastases, maximum tumor size ≤50 mm and only anterolateral (segment 2, 3, 4b, 5 and/or 6) or posterosuperior (segment 1, 4a, 7 and/or 8) tumors. Patients treated with multiple liver resections and/or ablation therapy were excluded.
